# A validation study of the Zanmi Lasante Depression Symptom Inventory (ZLDSI) in a school-based study population of transitional age youth in Haiti

**DOI:** 10.1186/s13031-020-0250-9

**Published:** 2020-02-28

**Authors:** Rupinder K. Legha, Margaret E. Gerbasi, Mary C. Smith Fawzi, Eddy Eustache, Tatiana Therosme, J. Reginald Fils-Aime, Giuseppe J. Raviola, Emmeline Affricot, Ermaze Louis Pierre, Yoldie Alcindor, Jennifer Severe, Katherine A. Boyd, David J. Grelotti, Sarah Darghouth, Andrew Rasmussen, Anne E. Becker

**Affiliations:** 1 Center for Health Services and Society, University of California, 10920 Wilshire Boulevard, Suite 300, Los Angeles, CA 90045 USA; 2 Department of Global Health and Social Medicine, Harvard Medical School, 641 Huntington Avenue, Boston, MA 02115 USA; 3 Mental Health and Psychosocial Support Services Program, Zanmi Lasante, Santo 18A, Crois-des-Bouquets, Haiti; 4 Partners In Health, 800 Boylston Street, Suite 300, Boston, MA 02199 USA; 5 Department of Psychiatry, University of Michigan, University School of Medicine, 1500 East Medical Center Drive, Ann Arbor, MI 48109 USA; 6 Colorado School of Public Health, University of Colorado Anschutz Medical Campus, 13001 East 17th Place B119, Aurora, CO 80045 USA; 7 Department of Psychiatry, University of California San Diego, 9500 Gilman Drive, La Jolla, CA 92093 USA; 8 Department of Psychiatry, Massachusetts General Hospital, 15 Parkman Street, Boston, MA 02114 USA; 9 Department of Psychology, Fordham University, 441 East Fordham Road, Dealy Hall 334, Bronx, NY 10458 USA

**Keywords:** Depression screening tool, Validation, School-based assessment, Global mental health, Haiti

## Abstract

**Background:**

The Zanmi Lasante Depression Symptom Inventory (ZLDSI) is a screening tool for major depression used in 12 primary care clinics in Haiti’s Central Plateau. Although previously validated in a clinic-based sample, the present study is the first to evaluate the validity and clinical utility of the ZLDSI for depression screening in a school-based population in central Haiti.

**Methods:**

We assessed depressive symptoms in a school-based sample of transitional age youth (18–22 years; *n* = 120) with the ZLDSI. Other mental health-related assessments included a modified Structured Clinical Interview for DSM-IV-TR Axis I Disorders (SCID) for current Major Depressive Episode, the Center for Epidemiologic Studies Depression Scale, and selected items adapted from the Global School-Based Health Survey mental health module. Diagnostic assignments of major depressive episode (MDE) were based on modified SCID interviews.

**Results:**

The ZLDSI demonstrated good overall accuracy in identifying current MDE (Area under the Curve = .92, 95% CI = .86, .98, *p* < .001). We ascertained ≥12 as the optimal cut-off point to screen for depression with a sensitivity of 100% and a specificity of 73.9%. In addition, the ZLDSI was associated with other measures of depressive symptoms, suggesting that it demonstrates construct validity.

**Conclusions:**

Study findings support that the ZLDSI has clinical utility for screening for depression among school-going transitional age youth.

## Background

Depressive disorders are the fourth leading contributor to years lived with disability (YLDs) globally [1]. Although effective treatments are available, human and other resource shortfalls in low- and middle-income countries (LMICs) constrain both case-finding and care delivery. Relatedly, a large majority of individuals do not access treatment for serious mental disorders in LMICs [2]. One strategy for using available resources more efficiently is to move specific tasks from professionally trained mental health workers, who are less available in LMICs, to those who have fewer qualifications but who receive specific skills-based training [3]. Through this process known as task sharing, non-specialist health care providers such as community health workers (CHWs) can effectively deliver empirically supported treatments for depression in low-resource settings [4, 5].

However, adapting diagnostic assessments for mental disorders for use by non-specialist clinicians and CHWs remains a significant clinical challenge. Because the expression of symptoms and distress may be shaped by local meanings, values, and socioeconomic conditions, depressive disorders often present with varying phenotypes across socially diverse populations [6–8]. Consequently, standardized questionnaires and screening tools developed for North American or Anglo-European practice settings can lead to case misclassification in populations where symptoms are experienced and expressed in different ways [9]. Moreover, the associated diagnostic frameworks are often incongruent with how patients communicate psychological distress in community-based or primary care settings, where the majority of mental health treatment is provided in LMICs. Without validation in the local context, the clinical utility of screening instruments, therefore, may be limited [10].

For these reasons, developing locally derived, culturally-valid tools for identifying individuals with mental disorders represents a high-priority research agenda for building mental health capacity in resource-limited settings [11]. To be effective and feasible, such assessment tools should incorporate local vernacular and idioms of distress to facilitate lay understanding and expression of illness experience [12, 13] and must be easy to administer by non-specialist providers in non-clinical settings. The Zanmi Lasante Depression Symptom Inventory (ZLDSI) is one such locally derived tool. The ZLDSI is a structured screening tool incorporating local expressions of distress related to depression in Haitian Creole. It was developed as a decision support tool for identifying and referring individuals with signs of depression in clinical and community-based settings in Haiti’s Central Plateau [14].

Available evidence from Haiti supports a high prevalence of depression, PTSD, and suicidal ideation [15–17]; emotional distress related to violence and life stressors [18–20]; and exposure to traumatic events related to the 2010 earthquake [21–23]. Emotional distress and mental illness are frequently communicated through idioms of distress; patients and families often pursue religious and traditional healing resources for mental illness [24, 25]. For those deciding to seek biomedical care, clinicians may perceive common idioms of distress as signs of physical, rather than psychological, suffering [25].

Following a catastrophic earthquake centered near Port-au-Prince, Haiti in 2010, Zanmi Lasante (ZL), a Haiti-based non-governmental organization (NGO), and its sister organization Partners In Health (PIH), a Boston-based NGO, expanded mental health care services in Haiti’s Central Plateau and Artibonite Valley. These organizations have worked together to provide health care in Haiti for over three decades and now serve a catchment area of over 1 million persons. In the context of these expanded services [26], ZL and PIH developed the ZLDSI as a screening tool to guide CHWs’ and other non-specialist providers’ clinical decision-making within a depression care pathway. CHWs identify individuals in the community with signs and symptoms consistent with major depressive disorder using the ZLDSI. Based on ZLDSI scores, they refer severe cases to psychologists and social workers in the clinical setting, while managing less severe cases in the community using interpersonal therapy adapted to the Haitian context [27].

The present study builds upon the findings of the initial development of the ZLDSI by validating it against established measures of depression in a non-clinical study population from the same region, with the goal of establishing an optimal cut-point for school-based screening. A post-hoc exploratory aim examined the clinical utility of our identified ZLDSI cut-point for encompassing respondents with affirmative responses to the ZLDSI item regarding suicide-related ideation and behavior (SRIB).

## Methods

### Study setting

The present study draws on data from the screening component of a school-based pilot intervention in Haiti’s Central Plateau to promote student mental health conducted in 2013. In addition to the baseline screening for depression, PTSD, and SRIB [18], sequential phases of the Teacher-*Accompagnateur* Pilot Study (TAPS) included training teachers to recognize and respond to students at risk for mental illness, assigning each student study participant to a teacher participant counterpart, and facilitating one on one meetings between these teacher and student study participant counterparts to support student participant navigation to available mental health services at ZL when appropriate [28, 29].

### Study sample

The study sample (*n* = 120) for the present study is the same sample as described elsewhere for the student study participants of the TAPS study [18]. The study population comprised students from four secondary schools located in Haiti’s Central Plateau and in the ZL catchment area. Study participants were randomly selected from a student register at each school (*n* = 33 to 41) based on study eligibility criteria, which included enrolled students ages 18 to 22 years. Of 121 eligible students who provided informed consent and enrolled, one later withdrew consent and was discontinued from the study, so the corresponding study data were discarded. The overall response rate was 82.8%, and the lowest response rate for the four schools was 78.0%.

### Procedure

After providing informed consent, study participants responded individually, in writing to a battery of self-report psychosocial assessments during a single proctored session at their respective schools. Clinician-investigators with local expertise in mental health diagnostic assessment subsequently interviewed each participant using an abridged Structured Clinical Interview for DSM-IV-TR Axis I Disorders [SCID-I; 30], modified for this study. A version of the SCID corresponding to DSM-5 diagnostic criteria was not yet available at the time of data collection. Translation and adaptation of the SCID interview as well as procedures for study diagnostic assignment of major depressive episode (MDE) is described elsewhere [18]. Clinician-investigators conducted the ZLDSI assessment after the SCID interview on the same day. The majority of these research interviews performed by clinicians were conducted with 2 days of the self-report assessments (85.5%) and all were completed within 15 days. Both the Institutional Review Board (IRB) of the Harvard Faculty of Medicine and the Zanmi Lasante Ethics Committee approved the study.

### Assessments

#### Translation

Haitian Creole is the primary language spoken by Haiti’s over 10 million residents. Self-report psychosocial study assessments were translated from English into Haitian Creole by a bilingual study investigator and then independently back-translated. Original and back-translated versions were then compared, reconciled, and adjusted by bilingual members of the study team to optimize idiomatic and readily comprehensible usage. We adapted portions of a French language version of the SCID (Ouellette, personal communication, March 1, 2013) by translating questions posed to study participants from French into Haitian Creole, while the instructions to clinician-interviewers were retained in French (see [18]). Because the ZLDSI was originally developed in Haitian Creole, as described below, it did not require translation; this version was retained for the study [14].

#### Center for Epidemiologic Studies Depression Scale (CES-D)

We assessed depressive symptomatology with the Center for Epidemiologic Studies Depression Scale (CES-D) [31], a 20-item Likert-style self-report assessment which was slightly modified for this study [18]. Responses are scored as 0 to 3, and a total sum score is calculated with possible values ranging from 0 to 60. The internal consistency reliability of the measure was good in our sample as measured by Cronbach’s alpha (.86) and comparable to reliability reported in other study samples [31, 32].

#### Global school-based health survey (GSHS)

We assessed additional depressive symptomatology as well as SRIB with 6 items drawn from core and expanded module content of the WHO’s Global School-based Health Survey (GSHS) [33, 34]. The GSHS, a modular self-report questionnaire developed for surveillance of health risk and health promoting behaviors among school-children, has been implemented in over 90 countries and translated into nearly 20 languages [35], including Haitian Creole [36]. However, no Haitian Creole version had been posted or published when study data were collected in 2013. Table 1 describes content based on GSHS items and coding used for the present study; these items described in the present study also each appear on the current official Haitian version of the GSHS and include the same response options.

**Table 1:** Characteristics of the sample (*n* = 120, unless otherwise indicated)

	Mean (SD) or % (*n*)
Age in years, mean (SD)	19.47 (1.37)
Gender, % female (*n*)	33.33% (40)
ZLDSI^a^, mean (SD)	9.01 (8.49)
SCID MDE Cases, % (*n*)	7.50% (9)
CES-D^b^, mean (SD)	22.75 (12.38) (*n* = 111)
During the past 12 months, how often have you been so worried about something that you could not sleep at night? (GSHS)^c^, mean (SD)	2.46 (1.08)
During the past 12 months, how often have you felt lonely? (GSHS)^c^, mean (SD)	2.22 (1.05)
During the past 12 months, how often have you had a hard time staying focused on your homework or other things you had to do? (GSHS)^c^, mean (SD)	2.76 (1.18) (*n* = 119)
SRIB^d^ in GSHS, % cases (*n*)	6.67% (8)
SRIB^e^ in ZLDSI, % cases (*n*)	13.33% (16)

^a^ Scores from the ZLDSI are based on the degree to which the respondent is bothered by 13 symptoms in the past 15 days. Responses are scored 0 to 3, and a total sum score ranges from 0 to 39

^b^ Scores from the CES-D are based on a 20-item self-report assessment, slightly modified for this study [18]. Responses are scored 0 to 3, and a total sum score ranges from 0 to 60

^c^ Response options were: Never = 1, Rarely = 2, Sometimes = 3, Most of the time = 4, and Always = 5

^d^ Suicide-related ideation or behavior (SRIB) identified on the GSHS was coded as present if there was an affirmative answer to one or more of the following questions as indicated by a “yes” response to the first two and any value > = 1 time in response to the third question:

1. During the past 12 months, did you ever seriously consider attempting suicide?

2. During the past 12 months, did you ever make a plan about how you would attempt suicide?

3. During the past 12 months, how many times did you actually attempt suicide?

^e^ SRIB identified by the ZLDSI coded as present if there was any affirmative answer to the statement “During the past 15 days, how many times have you had thoughts that you would be better off dead, or of hurting yourself in some way?” An affirmative answer was indicated by positive responses to any one of the following: some days (1–5 days), more than a week (6–9 days), almost everyday (10–15 days)

#### ZLDSI

The ZLDSI is a 13-item screening tool designed to assist identification and triage of patients with depression in clinic and community based settings in rural Haiti [14]. Written in the Central Plateau’s regional dialect of Haitian Creole, the ZLDSI includes items that refer to three local idioms of distress relevant to depressive symptoms (e.g., *Kalkile twòp*, or“Thinking too much”) as well as locally familiar idiomatic expressions that align with clinical signs and symptoms of major depression, and inquire about the degree to which the respondent is bothered by each symptom in the past 15 days. Examples of symptoms expressed in idiomatic language include *Preske pa pran gou nan fè aktivite*, (“You feel you’ve lost the taste for doing anything” as a proxy for anhedonia); and *Gen difikilte pou dòmi pran ou*, (“Having a hard time falling asleep” as a proxy for insomnia). These questions are read aloud to each respondent, who is oriented to four response options (“Not at all,” “For a few days, 1-5 days,” “More than a week, 6-9 days,” and “Almost every day, 10-15 days”). These options are read aloud as prompts as often as needed and the interviewer records each response, with its corresponding numeric score (from 0 to 3). A score is calculated by summing the numeric responses, with possible scores ranging from 0 to 39 [14]. The ZLDSI includes a single item intended to capture SRIB: “Thoughts that you would be better off dead or of hurting yourself in some way.” In the development study, the ZLDSI demonstrated good internal consistency reliability, construct validity, and concurrent validity when screening for depression in a mixed-age (ranging from 14–75 years old) clinical convenience sample. The aforementioned study reported a score of 13 and higher as the optimal cut-point for screening in depression cases (with a sensitivity and specificity of 85.4 and 50.9%, respectively) in a Receiver Operating Characteristic (ROC) analysis [14]. Since its initial implementation in 2013, Zanmi Lasante CHWs and other providers have administered the ZLDSI widely, but its clinical utility for depression screening outside of a clinic-based sample—more closely representing the population and setting for which it is intended–has not yet been assessed. Examination of the ZLDSI’s validity for depression screening in a school-based sample diminishes concern for bias introduced by potential differences in presentation and severity among individuals who are treatment-seeking.

#### Abridged structured clinical interview for DSM-IV-TR Axis I disorders (SCID-I)

We used an abridged version of the Mood Episodes module of the Structured Clinical Interview for DSM-IV-TR [30], adapted for this study, to ascertain presence of MDE. For example, we omitted questions pertaining to etiologic factors and postpartum onset; past episodes; and catatonic, melancholic, and atypical features. We also included a study-specific ratings sheet for clinician-interviewers to summarize and record additional information about their diagnostic impression. The primary basis for determining caseness was based on the clinician-investigator ascertainment that MDE was present; study diagnostic assignments were finalized by consensus of three or more study investigators after review of written ratings, narrative, and summary data recorded during this interview for any disqualifying or supporting data (for additional details, see [18]).

### Data analysis

#### Data management

Raw data were entered into an Excel file and verified; missing and double entered responses were identified and addressed as previously described [18]. All study participants responded to both ZLDSI and SCID-based interviews; thus, study diagnostic assignments were available for all participants. In addition, complete ZLDSI data were available for the entire study sample (*n* = 120). For construct validity analysis utilizing the CES-D, we excluded study participants missing more than two CES-D items (*n* = 9); for construct validity analyses using GSHS content, we excluded one participant missing a response for the corresponding GSHS item. Deviations from the study sample due to missing CES-D or GSHS data are noted in Tables 1 and 3.

#### Statistical analyses

Internal consistency reliability for the CES-D and ZLDSI was estimated using Cronbach’s alpha. ROC analysis was used to evaluate the ability of the ZLDSI to discriminate between respondents who were classified as MDE cases and non-cases based on study diagnostic assignments [18]. The area under the ROC curve (AUC), ranging from 0.5 (indicating a test with no diagnostic capacity) to 1.0 (perfect diagnostic accuracy), was calculated to estimate the overall diagnostic accuracy of the ZLDSI, using the SCID-based assessment of current major depressive episode as the gold standard [37]. We evaluated sensitivity and specificity across a broad range of possible ZLDSI scores to determine a clinically optimal cut-off point, including the previously established cut-off point identified for a clinic-based population. Given the ZLDSI’s primary function for case finding and triage decision support by CHWs and specialty mental health professionals, we prioritized sensitivity over specificity in detecting depression. Next, to test dimensional diagnostic validity, we used logistic regression to assess whether continuous increases in the ZLDSI were associated with depression and SRIB as assessed by the SCID-based interview and the GSHS, respectively. To examine construct validity of the cut-off point, we used t-tests and chi-square tests to examine associations between the ZLDSI and other measures of depression as well as SRIB. In addition, as a post-hoc analysis of frequencies, we examined how frequently participants who responded affirmatively to the SRIB item on the ZLDSI would also be screened in by our identified cut-off point by calculating the percentage of respondents with SRIB who scored above and below the cut-off point. All analyses were conducted using IBM SPSS 23.

## Results

### Descriptive data

Table 1 displays demographic characteristics and clinical characteristics of the study sample. The sample comprised participants with a mean age of nearly 19.5 years and two-thirds were male. Of particular note, 7.5% of respondents had received a study diagnostic assignment of MDE in the past month and 6.7% had affirmed SRIB by written self-report in response to the GSHS-based items. A higher percentage of participants (13.33%) had indicated SRIB in responding to the ZLDSI interview.

### Reliability

The internal consistency reliability of the ZLDSI in the present school-based study sample was good, as measured by a Cronbach’s alpha = .90, comparable to the reliability reported in the initial validation of the ZLDSI with a clinic-based sample (alpha = .89 [14]).

### Validation

**Fig. 1:**
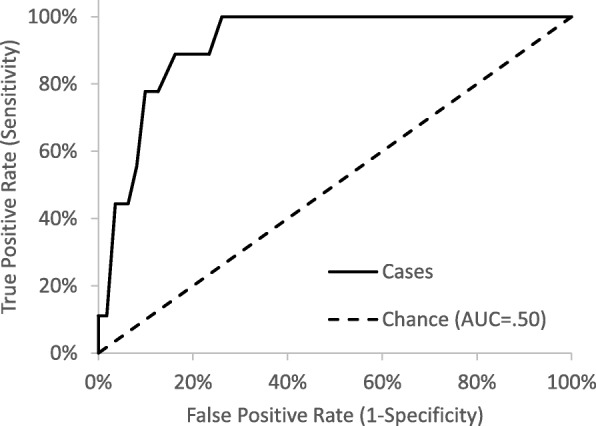
Receiver Operating Characteristic (ROC) Curve for the ZLDSI to Differentiate Major Depressive Episode Cases from Non-Cases Area Under the Curve (AUC) = .92, 95% CI = .86, .98, *p* < .001

Fig. 1 displays the ROC curve. Area under the curve (AUC) analysis supports that the ZLDSI demonstrated an overall accuracy of 92% (AUC = .92, 95% CI = .86, .98, *p* < .001). A ZLDSI score greater than or equal to 12 correctly identified 100% of cases in the sample and demonstrated a specificity of 73.9% (Table 2).

**Table 2:** Sensitivity and Specificity for SCID MDE Depression Diagnosis by ZLDSI Score (*n* = 120)

ZLDSI Score	Sensitivity	Specificity
5	100.00%	41.44%
6	100.00%	45.05%
7	100.00%	50.45%
8	100.00%	54.05%
9	100.00%	59.46%
10	100.00%	66.67%
11	100.00%	71.17%
12	100.00%	73.87%
13	88.89%	76.58%
15	88.89%	83.78%
16	77.78%	87.39%
17	77.78%	90.09%
18	66.67%	90.99%
20	55.56%	91.89%
22	44.44%	93.69%
26	44.44%	96.40%
31	11.11%	98.20%
36	11.11%	100.00%
39	0.00%	100.00%

### Dimensional validity

In our multivariable logistic regression using modified SCID-based MDE caseness as the dependent variable and adjusting for age and gender, a 1-point increase in ZLDSI score was associated with a 18% increase in the odds that a respondent had received a study diagnosis of MDE (AOR = 1.18, 95% CI = 1.08, 1.29, *p <* .001). Relatedly, a 1-point increase was associated with a 13% increase in odds that a respondent endorsed SRIB on the GSHS (AOR = 1.13, 95% CI = 1.04, 1.23, *p =* .002).

### Construct validity

Finally, to assess the degree to which the suggested cut-point of ≥12 on the ZLDSI resulted in identifying individuals experiencing greater depression symptomology, we tested the hypothesis that scores of 12 or higher on the ZLDSI would be associated with significantly greater levels of related psychopathology (i.e., as measured by the CES-D and selected items drawn from the GSHS) compared to those scoring 11 or lower. Comparison of group means and proportions via t-tests and chi-square tests demonstrated that the group with scores of 12 or higher showed significantly greater psychopathology across all indicators examined (Table 3).

**Table 3:** Relationship of ZLDSI and other measures of depression and suicidality (*n* = 120, unless otherwise indicated)

	ZLDSI < 12 (*n* = 82)	ZLDSI ≥12 (*n* = 38)	*p*
CES-D^a^, mean (SD), (*n* = 111)	17.72 (10.21) (*n* = 73)	32.41 (10.32) (*n* = 38)	<.001
During the past 12 months, how often have you been so worried about something that you could not sleep at night? (GSHS)^b^, mean (SD)	2.10 (.98)	3.24 (.88)	<.001
During the past 12 months, how often have you felt lonely? (GSHS) ^b^, mean (SD)	1.90 (.90)	2.89 (1.03)	<.001
During the past 12 months, how often have you had a hard time staying focused on your homework or other things you had to do? (GSHS) ^b^, mean (SD), (*n* = 119)	2.54 (1.11) (*n* = 81)	3.24 (1.22)	.003
SRIB in GSHS^c^, % cases (n)	0.0% (0)	21.15% (8)	<.001
SRIB in ZLDSI, % cases (n)	2.44% (2)	36.84% (14)	<.001

*p-*values correspond to independent sample t-tests for mean differences and Fisher’s exact tests for proportions

^a^ Scores from the CES-D are based on a 20-item self-report assessment, slightly modified for this study [18]. Responses are scored 0 to 3, and a total sum score ranges from 0 to 60

^b^ Response options were: Never = 1, Rarely = 2, Sometimes = 3, Most of the time = 4, and Always = 5

^c^ Suicide-related ideation or behavior (SRIB) identified on the GSHS was coded as present if there was an affirmative answer to one or more of the following questions as indicated by a “yes” response to the first two and any value > = 1 time in response to the third question:

1. During the past 12 months, did you ever seriously consider attempting suicide?

2. During the past 12 months, did you ever make a plan about how you would attempt suicide?

3. During the past 12 months, how many times did you actually attempt suicide?

Our post-hoc analysis of the clinical utility of this ZLDSI cut-off point of 12 to encompass individuals endorsing SRIB on the ZLDSI (*n* = 16) showed that whereas nearly all of the respondents with an affirmative response to this item had a ZLDSI total score above the identified cut point (*n* = 14), two of the participants in this subgroup had a score that fell below it, and thus would not have been identified based solely upon the ZLDSI cut-point.

## Discussion

Study findings support the clinical utility of the ZLDSI to identify depression in a school-based setting in Haiti’s Central Plateau. To our knowledge, the ZLDSI is the only brief screening tool for depression specifically developed for assessment of the population residing in Haiti’s Central Plateau. The present study is the first to examine the validity of different scores to detect cases of depression in a school-based setting, where the mental health burden is significant and treatment seeking for mental health care is limited [18]. These findings thereby support the validity of the ZLDSI when implementation is extended outside of a clinical setting, where this assessment tool can potentially promote detection of depression. By using structured (modified SCID-based) diagnostic assessment, our study also builds upon the initial validation study, which relied on a clinical assessment [14]; assessment of the ZLDSI against a gold standard diagnostic interview is a methodologically rigorous approach that further corroborates its validity as a screening tool for depression in this population [38].

Our findings support that a cut-off point of 12 optimized sensitivity and specificity in this school-based study sample. This was very similar to the cut-off point of 13 identified in a clinic-based convenience sample. However, we believe that the slightly lower cut-off point of 12 may be better suited to the identified clinical goals of high sensitivity and acceptable specificity in the Central Plateau community. Notably, the original screening development study reported similar sensitivity (89.6%) but less favorable specificity (47.4%) at a cut-off point of 12 in a sample of treatment-seeking Central Plateau residents [14].

Moreover, we argue the lower cut-off point of 12 offers clinically valuable sensitivity at the cost of modest losses in specificity relative to the originally proposed cut-off point of 13, which provided a sensitivity of 88.9% and specificity of 76.6% in our school-based sample and 85.4 and 50.9%, respectively, in the original clinical convenience sample. Underlying reasons for the disparate sensitivities and specificities across samples were not examined but may have been due to inherent differences between a population that is based in a clinical setting versus one that is not treatment-seeking. For example, if treatment seeking was partially driven by symptoms that overlap with neurovegetative signs of depression, respondents may have been more likely to endorse symptoms that resulted in a higher ZLDSI score [14]. Based on the aggregate findings in these two studies as well as the desirability of optimizing case-finding in the community, we suggest consideration of whether a cut-off point of 12 may offer optimal clinical utility in other non clinic-based settings in Haiti. Given our finding that the two respondents who endorsed suicidal ideation on the ZLDSI did not score above the cut-off point of 12, we further recommend active referral for services for any positive response to the ZLDSI suicidal ideation question, which is consistent with the current practice at ZL.

Our study has several limitations. Although we used a random sampling process within each of four schools to generate a school-based sample, the study population was not necessarily representative of the general population in Haiti’s Central Plateau for two reasons, given that it comprised a narrow age range and a school-going population. In fact, more than two-thirds of rural Haitian adults never attended secondary school, due to formidable social and economic barriers [39]. Next, our study evaluated the clinical utility of the ZLDSI in the hands of specialty mental health clinicians. We therefore suggest replicating this cut-off point in a community-based sample assessed by CHWs. We also recommend validating the ZLDSI in child and adolescent populations to establish its clinical utility for school and community-based screening among youth. Both could address significant case-finding gaps within the ZL/PIH community-based system of mental health care in rural Haiti. In addition, because the modified SCID and ZLDSI were performed by the same evaluator on the same day and relate to the same symptoms, SCID assessment could have influenced the ZLDSI. However, the ZLDSI is a structured questionnaire, during which the items are read verbatim and study participants respond to a closed ended set of options. Moreover, a ZLDSI score exceeding the cut-off point for depression is generated by summing values across 13 items rather than by applying clinical judgment to confer a diagnosis. These procedures would likely have reduced the potential for the SCID-based assessment to influence ZLDSI score. Finally, the strength of associations assessing construct validity may have been underestimated since there was a delay in administering the ZLDSI by more than 2 days following the self-report assessments with CES-D and GSHS items (for 14.2% of participants).

LMICs, which face a significant burden of disease related to mental illness but also lack adequate numbers of mental health specialists, require effective screening and assessment tools that perform well in the hands of CHWs and other non-specialist providers [11]. Unfortunately, the majority of existing brief screening instruments, developed primarily in Western countries, more accurately reflect mental health specialists’ understanding of mental disorders and patterns of phenomena observed in specialist settings, rather than the unique ways in which psychological suffering is expressed among general populations in community-based and primary care settings. Therefore, locally developed assessments—that follow a rigorous process for identifying and incorporating locally salient modes of expressing distress—are an essential tool in identifying individuals who could benefit from mental health care, but who may otherwise go undetected in biomedical settings [25, 40, 41]. Indeed, locally developed screening tools for common mental disorders may perform better in LMIC settings than standard measures do [38]. In order to diminish the global mental health treatment gap, tools like the ZLDSI are needed to address this “credibility gap,” or the gulf between mental health specialists’ understanding of mental disorders and how the rest of the world conceptualizes psychological suffering [10, 42].

The ZLDSI is brief, simple, and easy to use. It is an exemplar of a locally relevant assessment, given its development by drawing upon both idioms of distress and vernacular language that capture expressions of experiences and distress that align well with standardized, cross-national assessments of depressive symptoms like the SCID or CES-D [43]. A particular strength of the Zanmi Lasante/Partners In Health mental health services is its comprehensive approach, including a broad range of services such as culturally relevant psychoeducation for the wider community, psychosocial support for those in distress, and appropriate clinical services for those more severely affected with mental illness. Zanmi Lasante/Partners In Health’s efforts to develop school mental health services reflect such commitment to reducing the burden of mental disorders in Haiti. Zanmi Lasante is currently working with Ministère de la Santé Publique et de la Population, the Haitian Ministry of Health, toward scaling up community-based mental health care throughout Haiti. This scale-up would include the depression system of care, which relies upon the ZLDSI for case detection, triage, and symptom monitoring. The school-based pilot intervention, from which study data were drawn, is also being considered for scale-up, with the aim of increasing community-based identification of mental illness among youth. Therefore, validating the use of the ZLDSI as a screening tool for depression in Haiti in clinical and nonclinical settings has significant practical implications.

## Conclusion

Culturally valid assessments for depression are an essential tool for increasing local capacity to identify individuals who may benefit from mental health services in community settings. This expanded capacity is especially germane in a low-resource, post-disaster setting—such as in Haiti following the 2010 earthquake—in order to facilitate treatment access to meet needs associated with acute and chronic mental health burdens. Study findings support the validity of the ZLDSI for depression screening in a school-based setting in Haiti’s Central Plateau, building on and extending prior research on the ZLDSI in a clinical setting to support its clinical utility in a non-treatment seeking population.

## Data Availability

The dataset generated and analyzed during the current study is not publicly available in order to protect confidentiality of participants due to the relatively small study population defined by the selection criteria and the sensitive content of the data. The corresponding author will respond to reasonable requests to release a limited subset of the data, contingent on permission from the study co-PI (EE) and the two institutional IRBs with oversight.

## Abbreviations

AOR: Adjusted odds ratio
AUC: Area under the curve
CES-D: Center for Epidemiologic Studies Depression Scale
CHW: Community health worker
GSHS: Global School-based Health Survey
LMIC: Low- and middle-income countries
MDE: Major depressive episode
NGO: Non-governmental organization
PIH: Partners In Health
ROC: Receiver Operating Characteristic
SCID: Structured Clinical Interview for DSM-IV-TR Axis I Disorders
SRIB: Suicide-related ideation and behavior
TAPS: Teacher-*Accompagnateur* Pilot Study
YLD: Years lived with disability
ZL: Zanmi Lasante
ZLDSI: Zanmi Lasante Depression Symptom Inventory
